# Novel Molecular Weight Gradient Hyaluronate Dissolving Microneedles for Sustained Intralesional Delivery and Photodynamic Activation of Hematoporphyrin in Port-Wine Stain Therapy

**DOI:** 10.3390/polym17091238

**Published:** 2025-05-01

**Authors:** Xueli Peng, Chenxin Yan, Nengquan Fan, Chaoguo Sun, Suohui Zhang, Yunhua Gao

**Affiliations:** 1 Key Laboratory of Photochemical Conversion and Optoelectronic Materials, Technical Institute of Physics and Chemistry of Chinese Academy of Sciences, Beijing 100190, China; 2022111558@sdutcm.edu.cn; 2 Beijing CAS Microneedle Technology Ltd., Beijing 102609, China; yanchenxin@casmn.com (C.Y.); sunchaoguo@casmn.com (C.S.); 3 Research Institute of Marine Traditional Chinese Medicine (Qingdao Academy of Chinese Medical Sciences), Shandong University of Traditional Chinese Medicine, Qingdao 266112, China; 4 Chongqing Institute for Food and Drug Control, Chongqing 401121, China; fannqcq@163.com

**Keywords:** hematoporphyrin, molecular weight gradient design, dissolving microneedles, novel drug delivery, photodynamic therapy

## Abstract

Port-wine stain (PWS), a progressive congenital vascular malformation characterized by ectatic dermal capillaries, demonstrates age-dependent lesion expansion and chromatic intensification, resulting in significant psychosocial comorbidity. While systemic hematoporphyrin (HP) administration remains the clinical paradigm for photodynamic therapy (PDT), its therapeutic utility is severely constrained by non-targeted biodistribution. Pharmacokinetic analyses reveal prolonged dermal retention and suboptimal lesion accumulation, predisposing 42% of patients to phototoxic reactions. To address these limitations, this work creatively suggested a local targeted drug delivery method based on soluble microneedles in response to the difficulties mentioned above. The rational design of a molecular weight (MW) HA gradient system enabled the engineering of ternary nanocomposite microneedles with enhanced biomechanical integrity (0.49 N/needle) and superior HP loading capacity, which collectively facilitated spatiotemporally controlled transdermal delivery of hematoporphyrin with complete dissolution within 30 min. The release performance, skin permeability, and storage stability of hematoporphyrin dissolving microneedles (HP-DMNs) have all been demonstrated in vitro. This study applies soluble microneedle technology to the delivery of HP in PWS for the first time. It avoids the risk of systemic exposure through precise local administration. It uses the rapid dissolution properties of microneedles to achieve high concentration and rapid release of drugs in skin lesions. This study provides a new strategy for sustained intralesional release and rapid drug delivery treatment of PWS and provides novel ideas for the development of new formulations of HP and related photosensitizers.

## 1. Introduction

Pigmented vascular disease represents a congenital capillary malformation syndrome [1,2], predominantly manifesting as port-wine stains (PWS) and nevus flammeus lesions, which may occur with or without extracutaneous manifestations. Histopathological studies confirm that dermal capillaries undergo progressive ectasia, with lesions demonstrating significant size expansion and chromatic intensification during aging processes [3]. Approximately 30–40% of cases develop vascular hypertrophy and spontaneous hemorrhage, particularly in craniofacial regions [3]. These clinical progression patterns impose substantial psychological burdens on affected individuals.

As versatile theranostic agents, porphyrin derivatives have been extensively investigated for oncological interventions [4,5,6], photodynamic ablation [7,8], and fluorescence-guided surgery [9]. Hematoporphyrin (HP), a first-generation photosensitizer, exhibits superior photochemical reactivity and remains the most clinically utilized agent in photodynamic protocols [10]. Systemic intravenous injection of HP is a common application of photodynamic therapy in the clinical field [11]. Although photosensitizers have unique advantages, they have poor metabolic properties and lack specificity for lesions. Injection may induce severe systemic toxicity and side effects such as high fever and vomiting in patients, and regular daylight and even indoor lighting may cause complications.

Despite substantial research efforts on improving the therapeutic targeting and specific selection of HP, there are few attempts to combine new drug delivery systems with photosensitizers. At present, Yang et al. encapsulated HP in polymer micelles and added it to lactose microparticles as a pulmonary drug delivery system [12]. Xu et al. developed a degradable hyaluronic acid hydrogel system as a local delivery carrier for the photosensitizer protoporphyrin PpIX and the anticancer drug doxorubicin to achieve photodynamic combination therapy and on-demand drug release [13]. Huang et al. developed a tumor acidic microenvironment-activatable dissolving microneedle (DHA@HPFe-MN) to achieve controlled drug release and good melanoma chemo-photodynamic therapy through oxidative stress amplification [14]. As an emerging transdermal delivery platform, microneedle technology employs arrayed micro projections (500–1500 μm length) capable of bypassing the stratum corneum barrier with depth-tunable penetration [15]. Current microneedle systems are categorized into five archetypes: solid microneedles, coated microneedles, hollow microneedles, dissolvable microneedles, swollen microneedles, etc. [16]. Commonly used natural polymer materials for preparing microneedles include hyaluronic acid, polyvinyl alcohol, polyvinyl pyrrolidone, and chitosan [17].

The hematoporphyrin dissolving microneedle (HP-DMN) can deliver HP directly to the site of vascular malformations in the dermis, reducing the risk of systemic exposure and achieving precise delivery and targeted treatment. Microneedle local drug delivery can significantly reduce the accumulation of photosensitizers in non-target tissues, shorten the photoprotection period, and reduce systemic toxicity and photoprotection burden. Therefore, the HP-DMN system represents a paradigm shift in transdermal theranostics. To break through the limitations of traditional HP administration methods, it is a necessary attempt to develop HP-DMN preparations to achieve the precise treatment of PWS.

## 2. Materials and Methods

### 2.1. Materials

Hematoporphyrin API was purchased from Mylar Biopharmaceuticals (Chongqing, China); hematoporphyrin control was purchased from Med Chem Express (Shanghai, China); sodium hyaluronate (HA) was purchased from Bloomage Biotech (Beijing, China); ethyl cellulose (EC) and sodium carboxymethyl cellulose (CMC) were purchased from Aladdin Biochemical Technology Co. Ltd. (Shanghai, China); polyvinylpyrrolidone (PVP) was purchased from Boai Nky Pharma Co., Ltd. (Jiaozuo, China); injection-grade trehalose was purchased from Luofu Pharmaceutical Technology Co., Ltd. (Shanghai, China); sodium hydroxide was purchased from Enokai (Beijing, China); analytically pure hydrochloric acid was purchased from Beijing Chemical Factory (Beijing, China); phosphate buffer solution (PH 7.2–7.4) was purchased from Solepol Technology Co. Aldrich (St. Louis, MO, USA); ultrapure water (18.2 Ω, manufactured); chromatographically pure acetonitrile was purchased from Starco High Pure Solvents (Shanghai, China); analytically pure glacial acetic acid was purchased from Aladdin Biochemical Science and Technology (Shanghai, China); analytically pure Tween-80 was purchased from McLean Bio-technology Co. (Shanghai, China); and suckling pig skin was provided by Kaikai Science and Technology Trading (Shanghai, China);

Nude mice (male, 30 ± 2 g) were purchased from the Beijing Keyu Animal Breeding Center and the experiments were performed after 1 week of adaptive feeding. The animal research procedures were approved by the Animal Care and Use Committee of the Institute of Physical Chemistry, Chinese Academy of Sciences (Animal Experiment Ethics Number: IACUC-IPC-2501-001).

### 2.2. Compatibility Test of HP Excipients

To identify optimal polymer matrices for hematoporphyrin (HP)-loaded dissolving microneedles, we systematically evaluated the physicochemical compatibility between HP and five pharmaceutical-grade polymers: hyaluronic acid (HA, 240 kDa), polyvinylpyrrolidone K17 (PVP-K17), ethylcellulose (EC), carboxymethyl cellulose (CMC), and PVP-K90 (Table 1). For HA-based formulations, dissolve 0.02 g HP (1% *w*/*w*) in 1.78 g NaOH solution (0.2 M) using vortex mixing (2500 rpm, 25 °C, 5 min), titrate 45 μL HCl (5 M) at 2 μL/s under continuous agitation (Vortex-Genie 2, 20 min) to stabilize pH at 7.4 ± 0.1, incorporate 0.2 g HA (240 kDa) under light-protected conditions (lux < 5), and homogenize solution (500 rpm, 30 min) followed by 24 h quiescent storage at 25 °C. Then, all formulations were maintained: fixed HP loading (1% *w*/*w*), ionic strength (0.15 M NaCl equivalent), and ambient temperature (25 ± 0.5 °C). The viscosity of each matrix formulation was measured at 25 °C using an Anton-Paar ViscoQC300 series viscometer. In the steady-state shear test, the shear rate was increased from 0.1 to 100 s^−1^ and each gradient was balanced for 30 s to ensure data stability.

### 2.3. Establishment of High Liquid-Phase Analysis Method for HP

A high-performance liquid chromatography (HPLC) analysis method was developed for quantitative analysis of hematoporphyrin (HP) in dissolving microneedles. The procedure was as follows: the mobile phase consists of an aqueous phase and an organic phase, the organic phase is pure acetonitrile, and the aqueous phase solution is prepared by accurately weighing 200 mg of NaOH and 11.52 mL of glacial acetic acid in a 1L volumetric flask. Ultrapure water is used to make up the volume, and the solution is filtered using a 220 nm nylon membrane filter, and ultrasonic degassing is performed for 30 min.

Accurately weigh 2.00 mg HP in a 10 mL brown volumetric flask, add sufficient 90% acetonitrile solution to dissolve the sample, and after complete dissolution, dilute with the mobile phase to the mark to obtain a standard solution containing 200 μg/mL HP. Use mobile phase to dilute to obtain a gradient dilution solution containing 4–80 μg /mL HP. Accurately pipette 10 μL of each group of solutions for liquid-phase analysis and liquid-phase methodology establishment, and record spectral information such as peak area, theoretical plate number, and separation degree.

### 2.4. Preparation of HP-DMNs

The HP-loaded microneedle formulation was prepared through sequential solvent casting. The amount of 0.225 g hematoporphyrin (1.50% *w*/*w* final concentration) was dissolved in 12.950 g NaOH solution (0.2 M) via vortex mixing (2500 rpm, 25 °C, 5 min). A total of 270 μL HCl (5 M) was titrated incrementally (10 μL/min) under continuous vortex agitation (20 min) to achieve physiological pH 7.4 ± 0.2. Then, 0.150 g Tween 80 (1% *w*/*w*) was incorporated with 10 min magnetic stirring (500 rpm). Polymer matrix formation consisted of HA (240 kDa), HA (3 kDa), and trehalose, which was added in the order of 0.900 g high-MW hyaluronic acid (HA, 240 kDa, 6% *w*/*w*), 0.300 g low-MW HA (3 kDa, 2% *w*/*w*), and 0.420 g trehalose (2.80% *w*/*w*). After sequential addition, hydration was performed at 4 °C in the dark for 1 h.. The prepared substrate solution was dispensed (60 μL/well) into polydimethylsiloxane molds using an automated pipetting system (Eppendorf Xplorer, Hamburg, Germany). Vacuum-assisted drying was conducted under light-protected conditions to obtain crystallized microneedle arrays.

### 2.5. Morphological Observation and Puncture Performance of HP

The overall morphology of the HP microneedles was examined using a stereomicroscope (BA400, NIKON, Tokyo, Japan). The HP microneedles were sectioned into individual array strips to observe the fine structure of the needle tip. The puncture performance of the microneedles was evaluated using ex vivo piglet skin. Before the experiment, the surface moisture of the ex vivo pig skin was carefully blotted dry, and the skin was then laid flat on dry filter paper. A single microneedle array was affixed to the pig skin surface, and a needle inserter was employed to apply a force of 20 N/cm^2^ perpendicular to the skin surface for 20 s. An upright microscope was utilized to capture images of the microneedle array formed on the pig skin.

Subsequently, the skin samples were collected and fixed in 4% paraformaldehyde for 24 h. After fixation, the samples were embedded in paraffin, sectioned into 4 µm thick slices, and subjected to histological analysis using hematoxylin and eosin (H&E) staining.

### 2.6. Mechanical Properties of HP-DMNs

To evaluate the mechanical strength of the microneedles, tests were performed using a force stroke instrument in compression mode. Briefly, a single MN array (21 needles) was centrally positioned on the lower compression plate with needle tips aligned perpendicular to the upper platen. A stainless steel cylindrical sensor with a diameter of 10 mm was used and moved toward the needle tip at a speed of 0.2 mm/min to apply an axial force vertically to the microneedle until the test was completed. The force–displacement curve is recorded, and the fracture force is extracted.

### 2.7. Solubility Test of HP-DMNs

The HP microneedles were attached to the hydrogel backing, and a needle inserter was used to add a force of 20 N/cm^2^ to assist the microneedles in penetrating the ex vivo pig skin. The microneedles were removed after 5 min, 10 min, 20 min, and 30 min, respectively. The microneedles were cut into single array strips, and the dissolution was observed under a stereo microscope.

### 2.8. Distribution of HP-DMNs In Vivo

The spatiotemporal distribution of HP in nude mice (male, 30 ± 2 g) was longitudinally monitored using a calibrated in vivo imaging system (Photon Imager Optima, Biospace Lab, Nesles la Vallée, France). The prepared HP-DMN was applied to the back of nude mice, pressed, and applied for 30 min before being removed. The nude mice were anesthetized again at the corresponding time points of 0, 1 h, 2 h, 4 h, 6 h, 8 h, 24 h, and 48 h, and the mice were imaged in vivo. Fluorescence parameters were optimized based on the Soret band absorption and aggregation-specific emission profiles of HP, as reported in prior studies [1,10,18,19,20]. After pre-experimental spectral calibration, excitation/emission wavelengths were set to 537 ± 2 nm and 622 ± 5 nm, respectively, and the exposure time was 5 s. The distribution of HP in the back skin of nude mice from 0 to 48 h was recorded and expressed as mean ± SD (n = 5).

### 2.9. In Vitro Release Test of HP-DMNs

The in vitro release test (IVRT) of HP-loaded microneedles was systematically evaluated using a validated reciprocating cylinder dissolution system (USP Apparatus 3, DISSO III-7, LOGAN Instruments, Somerset, NJ, USA) under relevant conditions. A phosphate-buffered saline (PBS, pH 6.8 ± 0.1) medium was prepared by mixing 1020 mL 0.1 M NaH_2_PO_4_ and 980 mL 0.1 M Na_2_HPO_4_ to simulate dermal interstitial fluid. Each 250 mL dissolution vessel maintained 220 mL medium at 32.0 ± 0.5 °C (matching skin surface temperature) with reciprocation parameters set to 15 dips/min, 4 cm stroke length, and 10 mL continuous medium replenishment. Triplicate HP-DMN arrays were secured to cylindrical glass mounts using bioinert silicone adhesive (Dow Corning 732), ensuring full contact with hydrodynamic flow patterns. Aliquots (3 mL) were collected at 5, 10, 30, 120, 180, 240, and 360 min through 0.22 μm PVDF membrane filtration, with immediate iso-volumetric medium compensation. The quantification of HP release was performed via validated HPLC (Agilent 1260, Agilent Technologies, Inc., Beijing, China).

### 2.10. In Vitro Permeation Test of HP-DMNs

The in vitro permeation test (IVPT) of HP from dissolvable microneedles was conducted using a validated Franz diffusion cell system (modified ASTM D1708-13a [21]) with porcine skin. Epidermal membranes prepared with a 6 mm biopsy punch were hydrated in PBS (pH 7.4, 32.5 ± 0.5 °C) for 1 h prior to microneedle application. HP-loaded microneedles were inserted under controlled pressure (10 N·cm^−2^, 20 s). Mounted membranes were secured in vertical diffusion cells (SYT-101, Eddy Kuo Technology Co., LTD., Yanji, China, 1.48 cm^2^ effective area) with stratum corneum facing the donor chamber containing 4 mL PBS receptor medium (37.0 ± 0.5 °C water jacket, 300 rpm magnetic stirring). Full-volume sampling with iso-perfusion replacement occurred at 3, 6, 8, and 24 h, with aliquots filtered through 0.22 μm nylon membranes prior to HPLC-UV quantification (Agilent 1260, Agilent Technologies, Inc., Beijing, China).

### 2.11. Stability Test of HP Soluble Microneedles

To assess the storage stability of microneedle dry films, samples were hermetically sealed in aluminum/polyamide blister packaging (ISO 13485-compliant [22]) and subjected to accelerated stability testing under ICH Q1A(R2) guidelines. Quadruplicate batches were stored in controlled-environment chambers (TMT-200X, Zhujiang Taihongjun Instrument, China) at −20 °C (cryopreservation), 4 °C (refrigerated), 25 °C/60% RH (ambient), and 40 °C/75% RH (accelerated) for 21 days. Critical quality attributes were monitored at weekly intervals (days 7, 14, 21) through drug content stability.

## 3. Results and Discission

### 3.1. Compatibility Test Results of HP and Excipients

**Figure 1:**
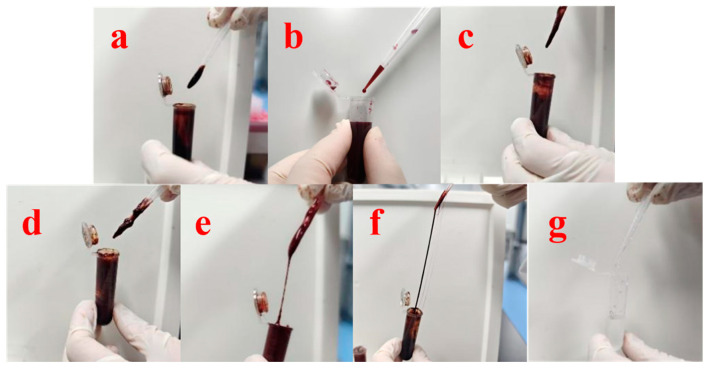
Compatibility of HP with commonly used polymer excipients. (**a**): HP+HA; (**b**): HP+PVP-K17; (**c**): HP+EC; (**d**): HP+CMC-8000; (**e**): HP+PVPK90; (**f**): HP+HA+PVPK90; (**g**): PVPK90.

The compatibility between HP and HA was relatively good (Figure 1a), with no HP precipitation, and the matrix fluid formed by HA was moderately viscous, while the matrix fluid formed by PVP-K17 was less viscous (Figure 1b). HP was found to be generally compatible with EC, as well as HP with CMC-8000 (Figure 1c,d), with the matrix fluid formed being more viscous, in which the CMC-8000 matrix fluid had a granularity and HP precipitation. The compatibility between HP and PVP-K90 was low. The matrix fluid formed was high in viscosity and showed pulling, which was initially speculated to be the cross-linking of HP and PVPK90 (Figure 1e). Similarly, with HA and PVP-K90 as excipients, a more pronounced pulling of the matrix solution occurred (Figure 1f), whereas with 10% PVP-K90 matrix solution alone, the solution was less viscous. The matrix solution did not show any pulling (Figure 1g), which further corroborated the cross-linking reaction between HP and PVPK90, and therefore, PVP-K90 was not considered to be added as a microneedle adjuvant in the subsequent tests. HA was selected as the HP microneedle excipient. The dynamic viscosity (η) of polymeric solutions was determined using a viscometer under steady-state shear conditions (shear rate range: 0.1–100 s^−1^, temperature: 25.0 ± 0.1°C). The measured values (mean ± SD, n = 3) were as follows: 25,500 ± 25 mPa·s for hyaluronic acid (HA, 240 kDa), 1,986 ± 17 mPa·s for polyvinylpyrrolidone K17 (PVP-K17), 13,090 ± 23 mPa·s for ethylcellulose (EC), 19,469 ± 13 mPa·s for carboxymethylcellulose (CMC-8000), and 12,890 ± 24 mPa·s for polyvinylpyrrolidone K90 (PVP-K90). The cross-linking reaction between polyvinylpyrrolidone K90 (PVPK90) and HP induced phase separation in the HA-PVPK90 composite solution, resulting in macroscopic inhomogeneity that precluded reliable rheological characterization of the matrix viscosity. This behavior may arise from competitive interactions between HA and PVPK90, where HP-mediated cross-linking of PVPK90 disrupts the homogeneous entanglement network, leading to thermodynamic incompatibility.

### 3.2. HPLC Analysis of HP-DMNs

To analyze the drug loading in HP-DMN, a liquid-phase analysis method dedicated to HP content detection was established. The chromatographic conditions were as follows: instrument: Agilent 1260, detector: UV, chromatographic column: ChromCore 120 C18, 5um, 4.6 × 250 mm, detection wavelength: 400 nm, flow rate: 1 mL/min, column temperature: 25 °C, and injection volume: 10 μL. The chromatographic conditions were set as gradient elution (Table 2).

### 3.3. Validation of Liquid-Phase Methodology

**Figure 2:**
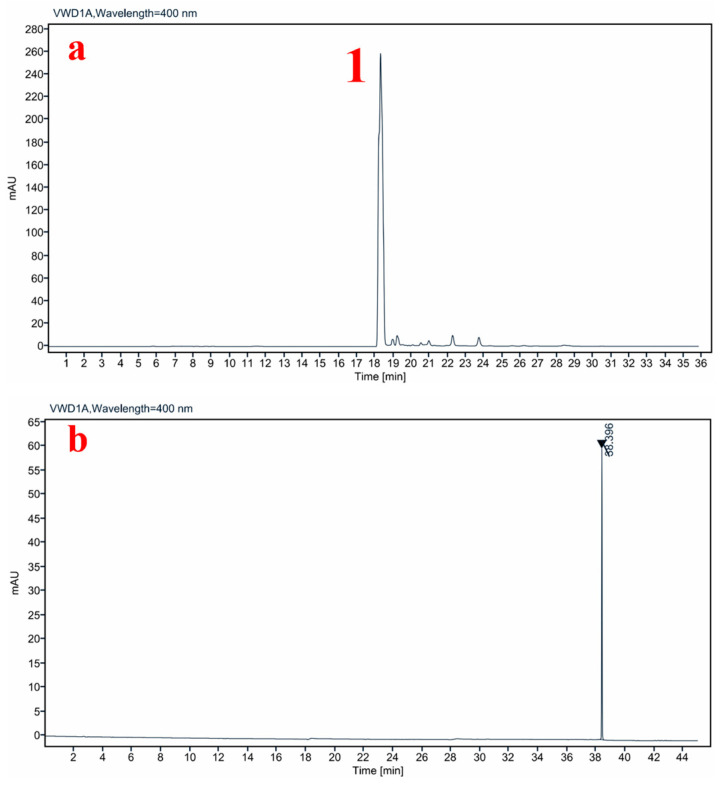
Specificity of liquid-phase conditions; (**a**) HPLC spectrum of HP-DMN, 1 is the target peak; (**b**) HPLC spectrum of blank microneedles.

The amounts of 10 μL of HP standard solutions with concentrations of 4–80 μg/mL were drawn for liquid-phase analysis. The goodness of fit R2 of the HP standard curve was one, and the linearity of the HP standard curve obtained by this analytical method was good. In addition, 10 μL of the standard solution containing 40 μg/mL HP was accurately drawn and repeated six times. The results showed that the RSD of the chromatographic peak area was 0.18%, and the repeatability of the HPLC analysis system applied to HP was good. In another way, blank microneedles without HP were prepared, dissolved in the mobile phase, filtered with a 0.22 μm organic filter, and then detected and analyzed by the same liquid-phase method. The results showed that the blank microneedles did not have any absorption peak at 18.3 min (Figure 2), indicating that the excipients involved in the formation of the microneedles would not interfere with the detection of HP, and the detection specificity of this liquid-phase method was excellent.

### 3.4. Microneedle Matrix Formulation Selection for HP-DMNs

**Figure 3:**
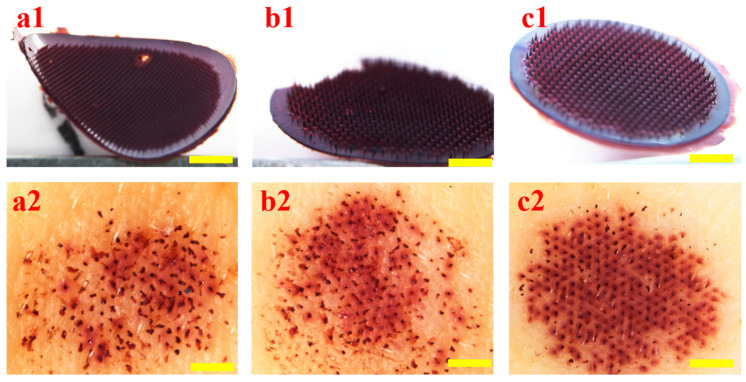
Microneedle formation and puncture properties of HA with different molecular weights: ((**a1**,**a2**) are 8% HA (240 kDa); (**b1**,**b2**) are 8% HA (3 kDa); and (**c1**,**c2**) are mixed matrices of 6% HA (240 kDa) and 2% HA (3 kDa); ×1, bar = 1 mm).

In the previous research (Figure 3), we found that the needle-forming conditions of HA matrix with a single molecular weight were poor, and there were some shortcomings: the microneedles made of high molecular weight HA (240 kDa) alone have worse penetration and are easily bent. The microneedles made of low molecular weight HA (3 kDa) alone have lower flatness and are prone to breakage. However, the addition of both high molecular weight HA (240 kDa) and low molecular weight HA (3 kDa) results in a smoother microneedle with better penetrability. After preliminary experiments, we concluded that 6% high molecular weight HA (240 kDa) can obtain good support and formability in microneedles, and adding a small amount (2%) of low molecular weight HA (3 kDa) can increase the hardness and mechanical strength of microneedles and improve the punctureability of microneedles.

### 3.5. Morphology and Puncture Performance of HP-DMNs

**Figure 4:**
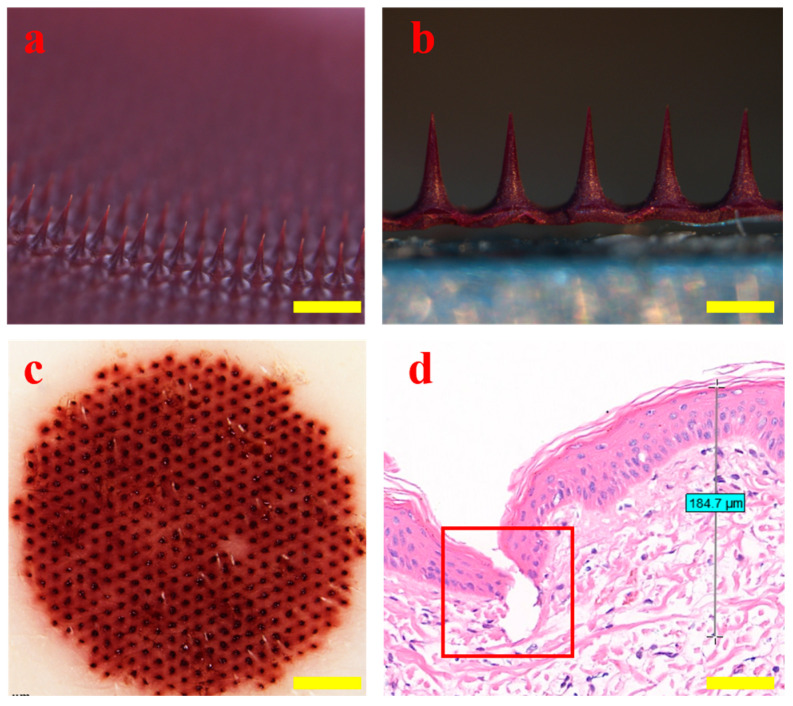
Morphology and puncture properties of HP-DMNs. ((**a**): ×2, bar = 500 μm; (**b**): ×5, bar = 200 μm; (**c**): ×1, bar = 1 mm; (**d**): ×10, bar = 0.1 mm).

The morphology of HP-DMN was observed using a stereo microscope (BX51, Olympus, Tokyo, Japan). The HP-DMN has a full body and no missing needle tip (Figure 4a,b). The puncture performance of the microneedle was tested using piglet skin. The HP-DMN with a needle height of 500 μm was inserted into the ex vivo piglet skin, and a clear array of pinholes was formed on the piglet skin (Figure 4c), indicating that the HP-DMN has excellent puncture performance. The HE-stained suckling pig skin tissue sections showed that the skin would wrinkle when the microneedle penetrated the skin. The penetration depth of the microneedle was about 184.7 μm. Therefore, HP-DMN could penetrate the stratum corneum of the skin and deliver the drug to the affected area of the dermis (Figure 4d).

### 3.6. Mechanical Strength of HP-DMNs

**Figure 5:**
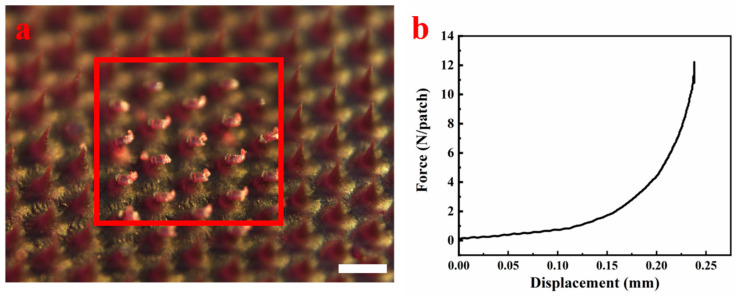
Mechanical strength of HP-DMNs. ((**a**) Schematic diagram of mechanical test microneedle, ×4, bar = 500 μm. (**b**) Force and displacement curve).

According to the results of the mechanical tests (Figure 5), the number of microneedle roots that could be accessed by the sensor plane was 21, and the force was about 10.52 ± 0.39 N (n = 3). According to the literature [23], if the force of a single microneedle exceeds 0.098 N, it is considered to have good mechanical strength. The force of a single needle in HP-DMN is about 0.50 ± 0.02 N, so it is believed that HP-DMN has excellent puncture performance and mechanical strength. The linear relationship between the total force and the number of needles indicates that the mechanical response of the microneedle array is uniform, and the single needle force is 0.50 N, far exceeding the skin penetration threshold of 0.098 N, suggesting that HP-DMN can effectively overcome the viscoelastic resistance of the skin. Compared with traditional PVP or HA-based microneedles (single needle force is usually 0.1~0.3 N), the higher strength of HP-DMN may result from the optimization of its polymer molecular weight.

### 3.7. Solubility Test Results of HP-DMNs

**Figure 6:**
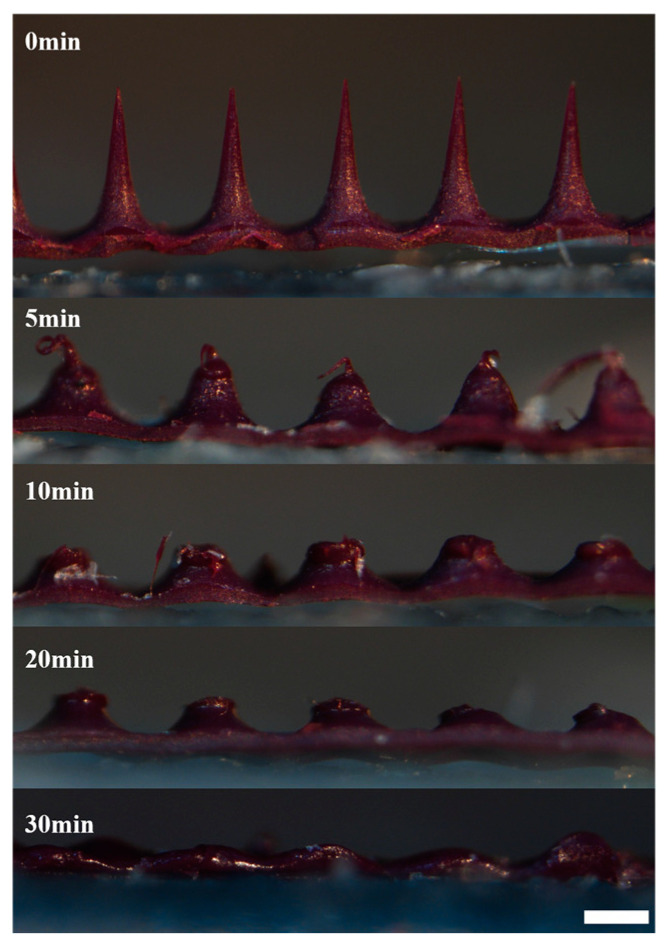
In vitro solubility of HP-DMN at various times (×5, bar = 200 μm).

HA is a natural polymer material with good biosafety [24]. HA has a strong hydration ability and can quickly combine with water and dissolve in the body [25]. HP-DMN dissolved more than 90% of the needle tip in 30 min (Figure 6). This shows that HP-DMN has excellent solubility properties, and the rapid dissolution of the microneedle tip can help the drug quickly enter the skin to take effect.

### 3.8. In Vivo Fluorescence Imaging of HP-DMNs

**Figure 7:**
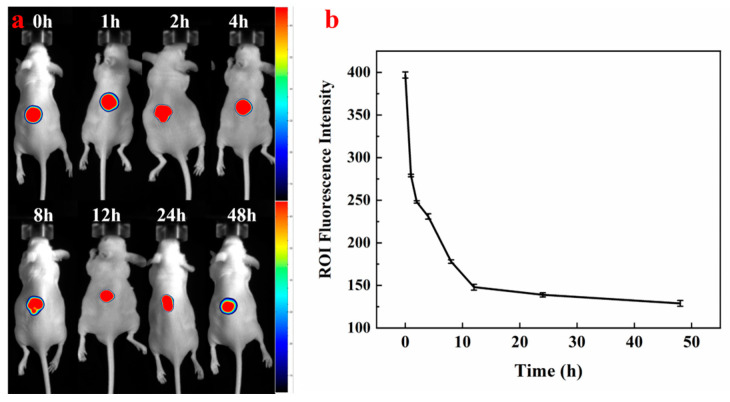
(**a**) Distribution, residence, and metabolism of HP-DMN in the skin of nude mice; (**b**) fluorescence intensity changes in HP-DMN in the skin of nude mice over time (n = 5).

The changes in fluorescence intensity in the in vivo imaging images can intuitively show the distribution, residence, and metabolic rate of HP-DMN on the skin surface (Figure 7). After 30 min of HP-DMN application, the microneedles dissolved in the skin and obvious fluorescence occurred at the administration site, indicating that HP had been delivered to the skin by the microneedles. With the increase in time, the fluorescence intensity declined (Figure 6), indicating that HP is in the process of metabolism within the skin.

### 3.9. In Vitro Release Test Results of HP-DMNs

**Figure 8:**
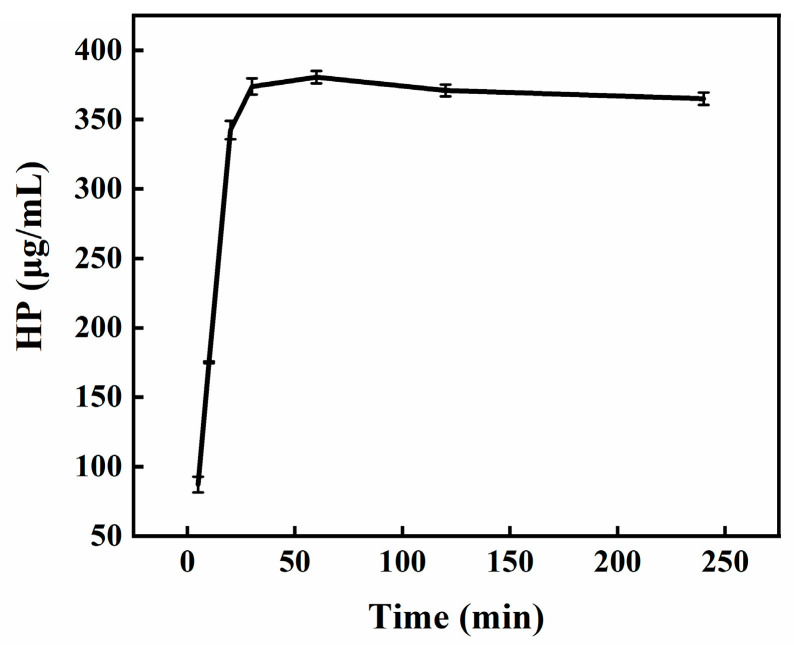
In vitro release curve of HP-DMN.

To test the drug release performance of HP-DMN, the reciprocating cylinder method was used for the experiment. HPLC detection showed that the average HP content in a single piece of HP-DMN was 381.87 ± 3.11 μg. The drug content measured by HPLC at 30 min was 342.2 ± 6.56 μg, which was about 89.61% of the total drug release. After that, the concentration in the solution tended to be stable, and the drug was basically released completely. It can also be intuitively seen from Figure 8 that HP-DMN showed a rapid release phenomenon within 0–30 min.

### 3.10. In Vitro Permeation Test Results of HP-DMNs

Franz diffusion cell is an internationally recommended method for evaluating in vitro drug permeation and release. It has the following advantages, such as simple operation, no need for continuous sample collection, and small amount of drug required for analysis [26]. This transdermal test used HP-DMN with a needle height of 500 μm as the test model. HPLC detection showed that the average HP content in a single piece of HP-DMN was 381.87 ± 3.11 μg. As shown in Table 3, only 8.36 μg of HP-DMN was permeated after 3 h, indicating that there was no burst release of HP-DMN. After 24 h, the cumulative percutaneous permeability was 20.07%. HP is a poorly soluble substance. The main method of drug delivery is intravenous injection. Microneedle preparations can improve the transdermal delivery efficiency of HP and provide a new direction for the delivery of HP.

### 3.11. Storage Stability of HP-DMNs

**Figure 9:**
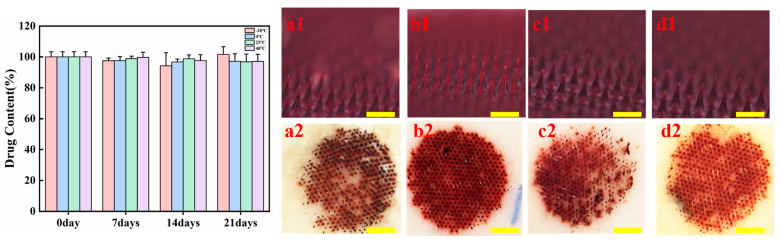
The stability results of HP-DMNs. The left figure shows changes in drug content of HP-DMNs over 21 days, and the right figure shows a, b, c, and d are the microneedle morphology and puncture performance under −20 °C, 4 °C, 25 °C, and 40 °C, respectively. ((**a1**–**d1**): Schematic diagram of microneedle morphology under different conditions after three weeks, ×4, bar = 500 μm; (**a2**–**d2**): Schematic diagram of microneedle puncture under different conditions after three weeks ×1, bar = 1 mm).

The packaged HP-DMNs were allocated into four storage condition groups for stability evaluation: (i) refrigerated storage at 4 °C, (ii) frozen storage at −20 °C, (iii) ambient conditions (25 °C, 30 ± 5% RH), and (iv) accelerated stability testing in a controlled environmental chamber (40 °C, 60 ± 5% RH). The changes in the content of HP in the microneedles were tested in the first week, the second week, and the third week (n = 7). The results showed that the content of HP-DMN did not change significantly within 3 weeks (Figure 9). After 3 weeks, HP-DMN still had good morphology and typical puncture properties, which indicates that the formula and microneedle form provided in this study can improve the storage stability of HP.

## 4. Conclusions

By screening the interaction between common polymer excipients and HP, the compatibility of five excipients—HA, PVP-K17, EC, CMC, and PVP-K90, with HP—was tested. Among them, HA had good compatibility with HP; no HP precipitation occurred in the solution within 24 h, and the solution was uniform and stable. For the first time, the molecular weight gradient HA formula is designed, and the synergistic effect of molecular chain entanglement and hydrogen bonding is achieved, which balances the excellent mechanical strength and rapid dissolution effect that traditional single molecular weight HA formula microneedles cannot achieve. HA is an acidic mucopolysaccharide with strong hygroscopicity and viscosity. It can fold to form a three-dimensional network structure to wrap HP and increase the stability of HP. A soluble microneedle array was successfully prepared by the vacuum filling method, with a puncture force of 0.51 N/needle, which can penetrate the ex vivo pig skin to the dermis (depth of about 184.7 μm), with excellent puncture performance and mechanical strength. The in vitro release test results showed that HP-DMN completely dissolved in simulated skin interstitial fluid within 30 min. In vitro dissolution tests showed that the microneedles completely dissolved in simulated skin interstitial fluid within 30 min, and the in vitro drug release reached 89.61% of the drug amount. The in vitro permeation test showed that after 24 h, the cumulative permeation rate was 20.07%. A highly sensitive HPLC detection method was developed, which had a good linear relationship in the range of 4–80 μg/mL, and the specificity and repeatability of the liquid-phase method were within the specified range. Although this study has made progress in the construction of the delivery system and stability optimization of HP-DMN, and HP-DMN has shown good puncture performance, solubility, and storage stability, the material of HP-DMN needs to be further optimized to increase the HP drug loading and photosensitization activity. In addition, pharmacokinetic and pharmacodynamic experiments, and photochemical characterization studies need to be continued to evaluate the safety, delivery efficiency, and long-term efficacy of microneedle delivery of HP. In summary, HP-DMN has the innovative potential of enhancing targeting, reducing side effects, and improving patient compliance in the treatment of PWS. The design of HP-DMN can provide new directions and an experimental basis for the development of new drug delivery routes for HP and its derivatives.

## 5. Patents

A patent has been issued based on the contents of this manuscript (Patent No. CN118717640A). The present invention provides a preparation type and preparation method of hematoporphyrin microneedle and relates to the technical field of biomaterials. This microneedle can improve the targeting effect of hematoporphyrin on the affected area and the drug utilization rate. And through this preparation method, the stability of hematoporphyrin can be improved and the local drug delivery concentration of the affected area can be increased.

## Figures and Tables

**Table 1:** Drug-excipient compatibility test table.

Group	Excipient	Added Content (%) *w*/*w*
1	HA	10
2	PVP-K17	10
3	EC	10
4	CMC	10
5	PVP-K90	10

**Table 2:** Gradient elution program of HP-DMNs.

Time (min)	Water Phase (%)	Acetonitrile (%)
0	55	45
15.00	55	45
15.10	20	80
25.00	20	80
25.10	0	100
35.00	0	100
35.10	55	45
45.00	55	45

**Table 3:** Percutaneous permeation test results of HP-DMN.

Time	Transit Dose (μg)	Total Transit Dose (24 h)	Total Transmittance (%)	Amount of Residue in Pig Skin (%)
3 h	8.36 ± 2.80	103.27	27.04	5.53
6 h	20.77 ± 6.63			
8 h	14.96 ± 3.24			
24 h	59.17 ± 2.67			

## Data Availability

Data are available on request due to restrictions, e.g., privacy or ethics; the data presented in this study are available on request from the corresponding author.

## Abbreviations

PWS	Port-wine stain
HP	Hematoporphyrin
PDT	Photodynamic therapy
MW	Molecular weight
HP-DMNs	Hematoporphyrin dissolving microneedles
HP-DMN	Hematoporphyrin dissolving microneedle
HA	Sodium hyaluronate
EC	Ethyl cellulose
CMC	Sodium carboxymethyl cellulose
PVP	Polyvinylpyrrolidone
PVP-K17	Polyvinylpyrrolidone-K17
PVP-K90	Polyvinylpyrrolidone-K90
HPLC	High-performance liquid chromatography
H&E	Hematoxylin and eosin
PVDF	Polyvinylidene fluoride
IVRT	In vitro release test
IVPT	In vitro permeation test

## References

[1] Lyu J., Wang S., Li Y., Zhang H., Yang Q., Liu S. (2022). Hematoporphyrin Monomethyl Ether-Mediated Photodynamic Therapy for Phakomatosis Pigmentovascularis Type II: A Case Report. Photodiagn. Photodyn. Ther..

[2] Diao P., Jiang X. (2023). Clinical application of photodynamic therapy in the treatment of port-wine stains. Bull. Dermatol..

[3] Chen J.K., Ghasri P., Aguilar G., van Drooge A.M., Wolkerstorfer A., Kelly K.M., Heger M. (2012). An Overview of Clinical and Experimental Treatment Modalities for Port Wine Stains. J. Am. Acad. Dermatol..

[4] Wang Y., Wei G., Zhang X., Xu F., Xiong X., Zhou S. (2017). A Step-by-Step Multiple Stimuli-Responsive Nanoplatform for Enhancing Combined Chemo-Photodynamic Therapy. Adv. Mater..

[5] Yu H., Yan Y., Chen X., Fan H., Yi M. (2022). Clinical application of hematoporphyrin photodynamic therapy for skin cancer. Jiangxi Med. J..

[6] Zhang F., Liu H., Su H., Ren J., Wang W., Wei H. (2022). Clinical study of hematoporphyrin-mediated photodynamic therapy combined with PD-1 inhibitor in the treatment of esophageal cancer. Chin. J. Laser Med..

[7] Wencheng L., Cho K., Yamasaki Y., Takeshita S., Hwang K., Kim D., Oda T. (2018). Photo-Induced Antibacterial Activity of a Porphyrin Derivative Isolated from the Harmful Dinoflagellate *Heterocapsa circularisquama*. Aquat. Toxicol..

[8] Yu H., Liu C., Ouyang C., Yan Y., Zhao S. (2024). Efficacy and safety of hematoporphyrin photodynamic therapy in the treatment of actinic keratosis. Jiangxi Med. J..

[9] Zhao T., Wu H., Yao S.Q., Xu Q.-H., Xu G.Q. (2010). Nanocomposites Containing Gold Nanorods and Porphyrin-Doped Mesoporous Silica with Dual Capability of Two-Photon Imaging and Photosensitization. Langmuir.

[10] Chang J.-E., Shim W.-S., Yang S.-G., Kwak E.-Y., Chong S., Kim D.-D., Chung S.-J., Shim C.-K. (2012). Liver Cancer Targeting of Doxorubicin with Reduced Distribution to the Heart Using Hematoporphyrin-Modified Albumin Nanoparticles in Rats. Pharm. Res..

[11] Chilakamarthi U., Giribabu L. (2017). Photodynamic Therapy: Past, Present and Future. Chem. Rec..

[12] Yang Y.-T., Chen C.-T., Yang J.-C., Tsai T. (2010). Spray-Dried Microparticles Containing Polymeric Micelles Encapsulating Hematoporphyrin. AAPS J..

[13] Xu X., Zeng Z., Huang Z., Sun Y., Huang Y., Chen J., Ye J., Yang H., Yang C., Zhao C. (2020). Near-Infrared Light-Triggered Degradable Hyaluronic Acid Hydrogel for on-Demand Drug Release and Combined Chemo-Photodynamic Therapy. Carbohydr. Polym..

[14] Huang Y., Lai H., Jiang J., Xu X., Zeng Z., Ren L., Liu Q., Chen M., Zhang T., Ding X. (2022). pH-Activatable Oxidative Stress Amplifying Dissolving Microneedles for Combined Chemo-Photodynamic Therapy of Melanoma. Asian J. Pharm. Sci..

[15] Le Z., Yu J., Quek Y.J., Bai B., Li X., Shou Y., Myint B., Xu C., Tay A. (2023). Design Principles of Microneedles for Drug Delivery and Sampling Applications. Mater. Today.

[16] Nagarkar R., Singh M., Nguyen H.X., Jonnalagadda S. (2020). A Review of Recent Advances in Microneedle Technology for Transdermal Drug Delivery. J. Drug Deliv. Sci. Technol..

[17] Ahmed Saeed AL-Japairai K., Mahmood S., Hamed Almurisi S., Reddy Venugopal J., Rebhi Hilles A., Azmana M., Raman S. (2020). Current Trends in Polymer Microneedle for Transdermal Drug Delivery. Int. J. Pharm..

[18] Li Y. (2022). Construction of Doxorubicin Prodrug/Hematoporphyrin Co-Assembled Nanoparticles and Investigation of Their Pharmacodynamics and Pharmacokinetics. Master’s Thesis.

[19] Zhao Y. (2024). A Preliminary Study of Hematoporphyrin Injection-Mediated Photodynamic Therapy Inducing Autophagy in 4T1 Cells of Mouse Mammary Adenocarcinoma. Master’s Thesis.

[20] Shao M. (2022). Experimental Study on Hematoporphyrin Derivative-Induced Lung Adenocarcinoma A549 Cells Transplantation in Nude Mice by Introducing 630nm Laser via Percutaneous Puncture. Master’s Thesis.

[21] (2013). Standard Test Method for Polymer-Crete Bonding Strength.

[22] (2016). Medical Devices—Quality Management Systems—Requirements for Regulatory Purposes.

[23] Demir Y.K., Akan Z., Kerimoglu O. (2013). Characterization of Polymeric Microneedle Arrays for Transdermal Drug Delivery. PLoS ONE.

[24] Tang L., Zhang Z., Lei S., Zhou J., Liu Y., Yu X., Wang J., Wan D., Shi J., Wang S. (2023). A Temperature and pH Dual-Responsive Injectable Self-Healing Hydrogel Prepared by Chitosan Oligosaccharide and Aldehyde Hyaluronic Acid for Promoting Diabetic Foot Ulcer Healing. Int. J. Biol. Macromol..

[25] Oh E.J., Park K., Kim K.S., Kim J., Yang J.-A., Kong J.-H., Lee M.Y., Hoffman A.S., Hahn S.K. (2010). Target Specific and Long-Acting Delivery of Protein, Peptide, and Nucleotide Therapeutics Using Hyaluronic Acid Derivatives. J. Control. Release.

[26] Salamanca C.H., Barrera-Ocampo A., Lasso J.C., Camacho N., Yarce C.J. (2018). Franz Diffusion Cell Approach for Pre-Formulation Characterisation of Ketoprofen Semi-Solid Dosage Forms. Pharmaceutics.

